# *Legionella pneumophila *pangenome reveals strain-specific virulence factors

**DOI:** 10.1186/1471-2164-11-181

**Published:** 2010-03-17

**Authors:** Giuseppe D'Auria, Nuria Jiménez-Hernández, Francesc Peris-Bondia, Andrés Moya, Amparo Latorre

**Affiliations:** 1 CIBER en Epidemiología y Salud Pública (CIBERESP), Barcelona, Spain; 2 Area de Genómica y Salud, Centro Superior de Investigación en Salud Pública (CSISP), (Avda. de Cataluña, 21), Valencia, (46020), Spain; 3 Instituto Cavanilles de Biodiversidad y Biología Evolutiva, Universitat de València, (Poligono La Coma s/n), Paterna, Valencia, (46071), Spain

## Abstract

**Background:**

*Legionella pneumophila *subsp. *pneumophila *is a gram-negative *γ-Proteobacterium *and the causative agent of Legionnaires' disease, a form of epidemic pneumonia. It has a water-related life cycle. In industrialized cities *L. pneumophila *is commonly encountered in refrigeration towers and water pipes. Infection is always via infected aerosols to humans. Although many efforts have been made to eradicate *Legionella *from buildings, it still contaminates the water systems. The town of Alcoy (Valencian Region, Spain) has had recurrent outbreaks since 1999. The strain "Alcoy 2300/99" is a particularly persistent and recurrent strain that was isolated during one of the most significant outbreaks between the years 1999-2000.

**Results:**

We have sequenced the genome of the particularly persistent *L. pneumophila *strain Alcoy 2300/99 and have compared it with four previously sequenced strains known as Philadelphia (USA), Lens (France), Paris (France) and Corby (England).

Pangenome analysis facilitated the identification of strain-specific features, as well as some that are shared by two or more strains. We identified: (1) three islands related to anti-drug resistance systems; (2) a system for transport and secretion of heavy metals; (3) three systems related to DNA transfer; (4) two CRISPR (Clustered Regularly Interspaced Short Palindromic Repeats) systems, known to provide resistance against phage infections, one similar in the Lens and Alcoy strains, and another specific to the Paris strain; and (5) seven islands of phage-related proteins, five of which seem to be strain-specific and two shared.

**Conclusions:**

The dispensable genome disclosed by the pangenomic analysis seems to be a reservoir of new traits that have mainly been acquired by horizontal gene transfer and could confer evolutionary advantages over strains lacking them.

## Background

*Legionella pneumophila *is a gram-negative facultative intracellular pathogen, identified as the infectious agent of the Legionnaire's disease (LD) or Legionellosis in 1977 [1]. It is found in aquatic environments parasitizing its natural hosts, amoebae and protozoa. From this environment, *Legionella *can colonize water treatment plants, such as refrigeration towers, potable water pipes, etc., and can cause infections in humans, when infected aerosols are inhaled [2,3]. Despite efforts to keep water systems free of *Legionella*, this pathogen is still causing infection throughout the world, including Spain, where it is endemic in some areas. From 1989 to 2005, around 310 outbreaks with 2,974 cases were recorded worldwide. In 2002 and 2005 there were two important epidemic events with 1,461 and 1,292 cases respectively. In Alcoy, an industrial town in the Valencian Region (Spain), a large outbreak occurred during 1999-2000. A strain that had caused several outbreaks and many cases, named "Alcoy 2300/99", was isolated from a patient in that outbreak [4]. Since then, recurrent epidemics in Alcoy have harbored Alcoy 2300/99.

Currently, the genomes of five *L. pneumophila *strains are available: Philadelphia (Lpg, USA) [5], Lens (Lpl, France) and Paris (Lpp, France) [6], Corby (Lpc, England) [7] and Alcoy (Lpa, Spain) (reported in this work). As with the majority of other pathogenic *Legionella *strains, immunoassay analysis defined them as belonging to the serogroup 1 [8]. A phylogeny based on Multi Locus Sequence Typing (MLST) showed that all strains are closely related, Alcoy and Corby being the closest [9].

Several features relating to the virulence of *L. pneumophila *are well known. For example, the mechanisms responsible for entry into the macrophages [10,11], the intracellular (host) trafficking of effectors [12] and the membrane-associated protein involved in virulence [9,13]. The data available disclose an almost complete physiology of this organism and its relationships with protozoa and human macrophages. An interesting question relating to *L. pneumophila *is its high rate of DNA exchange, not only within species and other closely-related bacteria, but also with eukaryotic organisms [14]. Comparative genomics can give clues about the extent of this process. Nowadays, the genome sequencing of strains belonging to the same species offers the possibility of defining their pangenome, which helps in understanding the evolutionary dynamics of microbial species. The pangenome comprises the core-genome, made up of the genes shared by all strains, and the accessory or dispensable genome compartment, consisting of the genes that are strain-specific or shared by only some of the strains [15]. Pangenome studies can disclose characteristics that are not easily perceptible using standard annotation analysis [16]. For example, pangenome studies have facilitated identification of virulence factors or anti-drugs systems in *Escherichia coli *and *Streptococcus agalactiae *[17,18]. The dispensable genome compartment can provide evidence of lateral gene transfer events that have occurred during the evolutionary history of a strain, probably offering additional evolutionary potential to the organism.

In this work, we report the main genomic features of *L. pneumophila *strain Alcoy 2300/99 and compare it with the four previously sequenced strains. A detailed description of the *L. pneumophila *pangenome is provided, and strain-specific features are catalogued in terms of "islands". Several islands containing virulence factors were identified and, where possible, their evolutionary origins were also hypothesized. Although the strains are phylogenetically closely related, the pangenomic approach allowed identification of distinctive features, such as anti-drug related islands, strain-specific transport or secretion systems, DNA transfer-related islands, CRISPR (Clustered Regularly Interspaced Short Palindromic Repeats) systems, and integrated phage insertions.

## Results and Discussion

### General features of the *L. pneumophila *Alcoy (Lpa) genome

A total of 36,974 and 215,350 sequences were obtained by the Sanger method (with an average length of 781 nt) and 454-technology (with an average length of 242 nt), respectively. The contigs were finally assembled in one continuous strand, with average consensus coverage of 22.1 ×. The Lpa chromosome length was of 3,516,335 base pairs, and 3,197 open reading frames were identified. The GC content was 38% and 1,175 predicted proteins were found to have unknown functions, representing 36.7% of all CDSs. Similar to Lpc and Lpg genomes, no plasmids were found in Lpa. Table 1 summarizes the main features of this genome compared to the other four *L. pneumophila *sequenced strains.

**Table 1:** Main features of *L. pneumophila *genomes

Features	Alcoy (Lpa)	Corby (Lpc)	Philadelphia (Lpg)	Paris (Lpp)	Lens (Lpl)
Accession Number	CP001828	NC_009494	NC_002942	NC_006368	NC_006369
Serotype	I	I	I	I	I
Genome length (bp)	3,516,335	3,576,470	3,397,754	3,503,610	3,345,687
Plasmid	0	0	0	1	1
GC content (%)	38.38	38.48	38.27	38.37	38.42
Coding genes (%)	86	86	88	87	86
Ribosomal operons	3	3	3	3	3
Islands bp (%)	157,442 (4.48)	217,089 (6.07)	180,555 (6.13)	163,637 (4.67)	180,986 (5.40)
Average GC of islands (%)	37.75	37.43	36.01	38.17	37.66

### Pangenome of *L. pneumophila*

**Figure 1:**
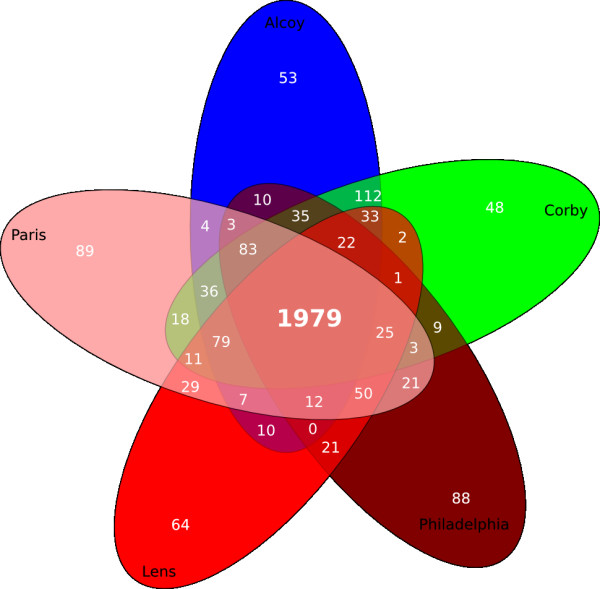
**Pangenome overview**. The Venn diagram showing the core genome and the genes specific of the strains *L. pneumophila *Alcoy, Philadelphia, Lens, Paris and Corby. Genes overlapping at least 70% length and 80% of similarity were considered orthologs.

Figure 1 summarizes the results obtained from comparing the five complete genomes of pathogenic *L. pneumophila *strains in relation to the orthologs/accessory gene distribution. The pangenome consists of 2,957 CDS with a core of 1,979 genes (66.9%) and a dispensable genome of 978 CDSs (33.1%). A total of 342 genes were found to be specific to the Lpa (53), Lpc (48), Lpg (88), Lpl (64) and Lpp (98) genomes. It is worth mentioning that 287 out of these 342 accessory genes (83.9%) were hypothetical proteins.

Lpa and Lpc are the strains that share most genes; 2,560 out of 3,196 in Lpa (80%) and 3,207 in Lpc (79.8%). This result is in agreement with the phylogenetic tree obtained using MLST [9]. Compared with the remaining genomes, Lpa and Lpc share 2,208 (69.1%) and 2,181 (68%) genes with Lpl; 2,271 (71.1%) and 2,284 (71.2%) with Lpp; and 1,802 (56.4%) and 1,776 (55.4%) with Lpg, respectively. Similarly, Lpp and Lpl also seem to be close-related with a shared genome of 2,207 genes out of 2,877 for Lpl (76.7%) and 3,026 for Lpp (73%). Finally, Lpg seems to be the most distantly related, sharing 2,207 genes with Lpl (75%) and 1,792 (60.1%) with Lpp.

**Figure 2:**
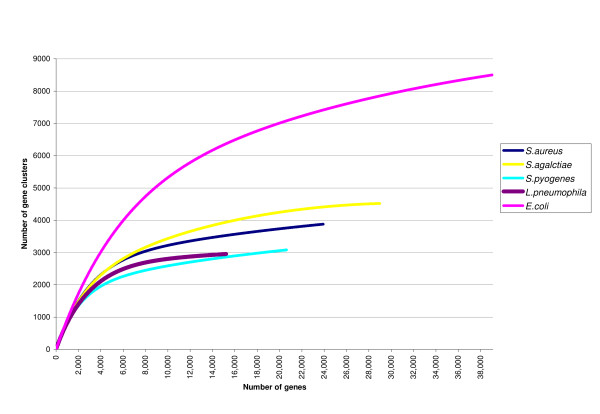
**Pangenome state**. Rarefaction curves applied to different strains of *L. pneumophila *(5 genomes), *E. coli *(8 genomes), *Streptococcus pyogenes *(8 genomes), *S. agalactiae *(8 genomes), *and Staphilococcus aureus *(9 genomes). See Additional file 6 for accession numbers of used genomes.

Figure 2 shows the application of the rarefaction methodology on the gene clusters from multiple genomes belonging to the same species. *L. pneumophila *tends to reach a plateau, although, according to Tettelin and collaborators [18], it should be considered an open pangenome similarly to what happens in other pathogenic organisms, such as the pangenomes from the same species of *Streptococcus agalactiae, S. pyogenes *and *Staphylococcus aureus *[18,19]. In the case of *E. coli*, despite the growing number of complete genomes, its pangenome is still far from fully described [17]. It has also been reported that clinically related pathogenic bacteria posses a lower level of variation than free-living bacteria, which is probably due to niche restriction that could lead to a wider core genome [20,21].

### Functional classification of core and dispensable genes

**Figure 3:**
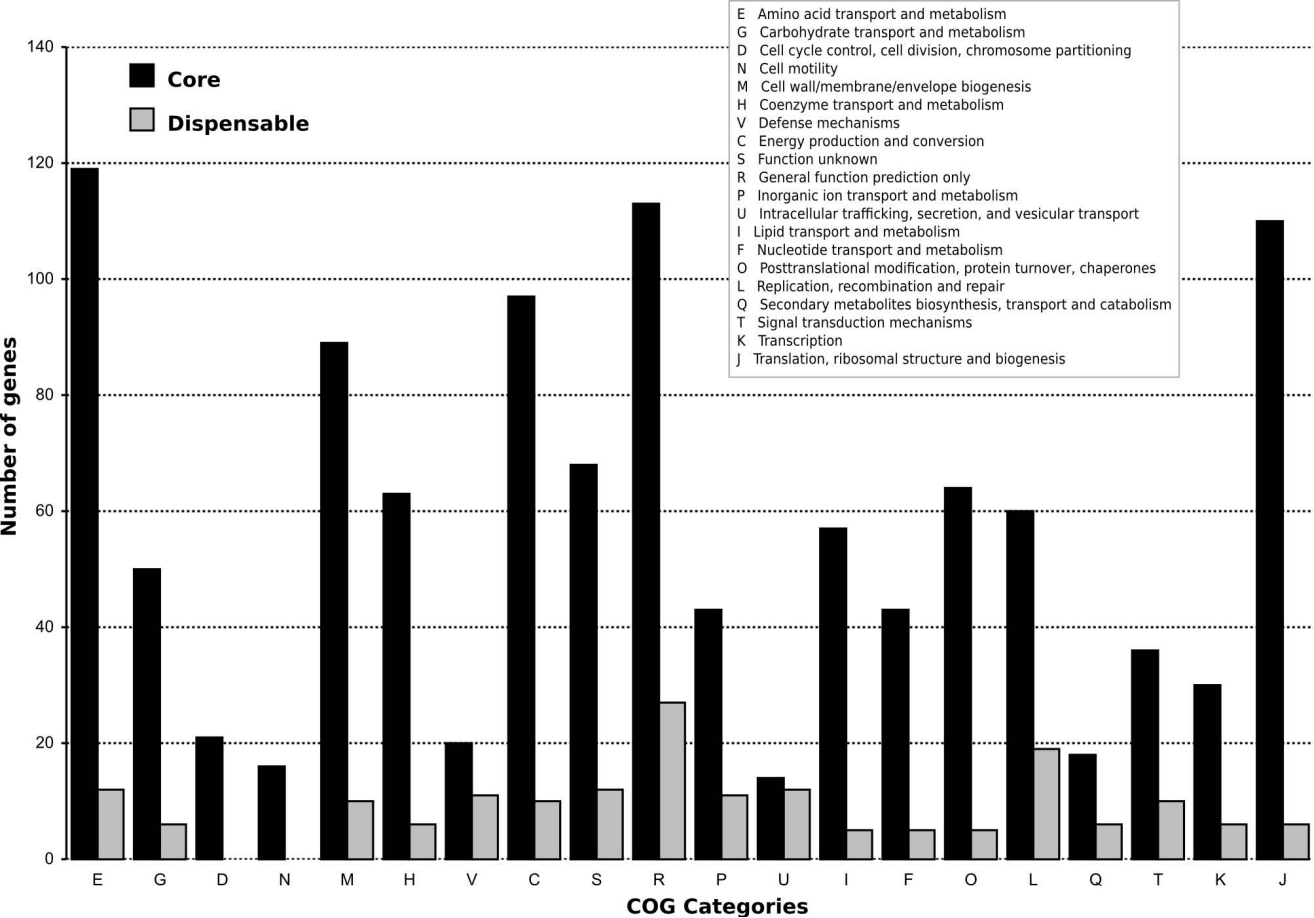
**Functional analysis**. *Functional analysis*. COGs distribution within the core and dispensable compartments of *L. pneumophila *pangenome.

Genes belonging to the core and dispensable genomes have been classified according to their predicted function based on COG categories (Figure 3). *L. pneumophila *is characterized by quite a high number of hypothetical proteins, for which annotation is still incomplete. Of the 1,979 genes belonging to the core genome, 1,131 (57%) were attributed to a COG category (*e *value less than 10^-15^), and in the case of dispensable genome, only 179 out of 978 (18.2%). These results are in agreement with those obtained in other studies, where hypothetical genes, and even genes with unknown function are, in the majority, in the dispensable genome [18]. Although the major proportion of the CDSs for which a function could be predicted (according to the COG database) falls within the core genome, minor differences between the two compartments were observed for defense mechanisms (V) and intracellular trafficking, secretion and vesicular transport (U) categories.

### Genomic islands

**Figure 4:**
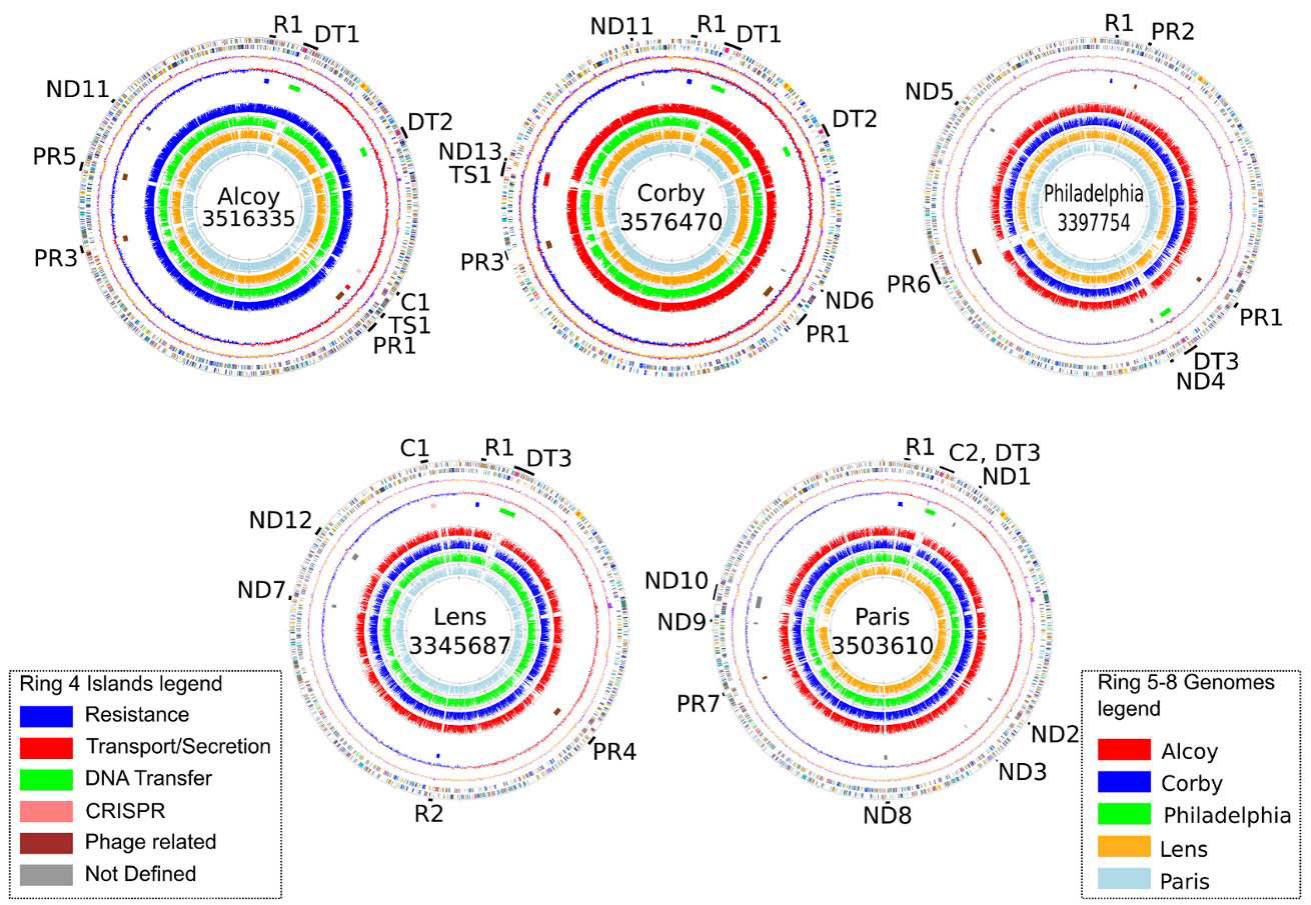
**Strain related orthology maps**. Strain related orthology maps. Each map shows comparative analysis based on each genome. From outside to inside: ring 1, gene positions according with COG categories color code; ring 2, GC content; ring 3, GC Skew; ring 4, island positions (color code according with islands legend); rings 5 to 8 show the BLAST homology for each gene versus the other four genomes (color code according with genomes legend).

**Figure 5:**
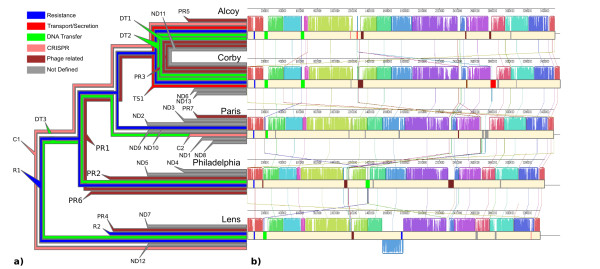
**Islands distribution on *L. pneumophila *MLST tree topology from D'Auria et al. (2008) **[9]. The left side (a) shows the islands appearance according to the tree topology obtained by the phylogenetic analysis. Each color is related to island type (see legend). On the right side (b) is reported the multiple genome alignment obtained by Mauve software applying default parameters [59]: homolog blocks are drawn with identical colors in forward and reverse strands; diagonal lines connect homologous blocks from each genome; colored blocks (according to island-type) on the light pink area between strands indicate the position of each island.

Although the five strains are highly syntenic, most of the genes that do not belong to the core genome are part of genomic islands, absent in at least one of the genomes. Table 2 reports the islands identified for each strain, Figure 4 describes the island positions for each genome following Table 2 classifications, while Figure 5a shows the hypothesis of islands histories according to the MLST tree topology obtained by D'Auria et al. [9]. Figure 5b shows the alignment of the five genomes, the locally collinear blocks and the position of the islands according to their genome locations. Twenty-eight islands were identified belonging to six different types (see Additional File 1). Only one island (R1, see below) is present in all five genomes; eighteen are strain-specific, probably acquired by horizontal gene transfer (HGT) events; five are common to Lpa/Lpc genomes (probably acquired by the Lpa/Lpc common ancestor), whereas one island (DT3, see below) could be interpreted as having been lost in the common ancestor of Lpa/Lpc genomes.

**Table 2:** Accessory genes islands

Island type	Genome	Details	Position	# of CDSs	Length	Transposase	GC %
*Resistance related islands (R)*							
	Alcoy	Contains several multi-drug related protein	68252 - 87883	22	19632	+	3534
	Corby		69153 - 88336	22	19184	+	35.30
	Philadelphia		68096 - 77398	9	9303	+	33.96
R1	Paris	Antibiotic persistance related system *HipB/HipA; Phage related proteins Xre, and multi-drug efflux pump*	66766 - 85124	21	18359	+	38.05
	Lens	Methylase, prophage integrase, TraK homologous	68898 - 83207	10	14310	+	36.77
R2	Lens	Stability system *StbDE*	1755441 - 1767402	10	11962	+	40.83
*Transport/secretion systems(TS)*							
	Alcoy	Cobalt/zinc/cadmium efflux transporter *helABC; ArsR *regulatory proteins	1251900 - 1268281	16	16382	+	40.75
TS1							
	Corby	Cobalt/zinc/cadmium efflux transporter *helABC*	2781725 - 2832225	56	50500	+	39.40
*DNA transfer (DT)*							
	Alcoy		181578 - 230836	61	49259	-	38.56
DT1		*tra *and *trb *conjugal transfer proteins, *Rac *integrase roteins, *htpX *protease prophage regulatory protein *alpA*					
	Corby		181312 - 241161	63	59850	-	39.11

	Alcoy		609680 - 648761	40	39082	-	39.52
DT2		Putative RNA helicase, two putative restrictases, *tra *and *trb *conjugal transfer proteins.					
	Corby		613277 - 656674	42	43398	-	39.25

	Paris	Plasmid-like elements containing *lvh*, *lvr*	183831 - 234043	45	50213	-	39.90
DT3	Philadelphia		1353613 - 1400796	45	47184	+	37.47
	Lens		172914 - 239494	64	66581	-	39.13
CRISPR systems (C)							
	Lens		3226572 - 3248046	16	21475	-	40.75
C1		Really similar CRISPR system					
	Alcoy		1169086 - 1179252	6	10167	-	38.49

C2	Paris	*Part of above P2 island	..	*45	....	....	....
*Integrated phage related (PR)*							
	Alcoy	MviN virulence factor	1292822 - 1329842	30	37021	+	36.14
PR1	Corby		1278158 - 1324830	49	46673	+	36.23
	Philadelphia		1167775 - 1185079	15	17305	+	37.33
PR2	Philadelphia	Mainly transposases	173401 - 183804	10	10404	+	35.14

	Corby		2493848 - 2532367	11	38520	+	38.11
PR3		Ankyrine containing domain					
	Alcoy		2486005 - 2509743	6	23739	+	37.00

PR4	Lens	Plasmid maintenance killer/antidote system	1190582 - 1219661	30	29080	+	34.79
PR5	Alcoy	Bacilysin system	2756698 - 2784130	33	27433	+	36.78
PR6	Philadelphia	Type IV secretion system	2296937 - 2366483	64	69547	+	38.04
PR7	Paris	Probable phage integration	2408503 - 2419758	16	11256	-	36.62
*Not well defined (ND)*							
ND1	Paris	Cytochrome *o*	325750 - 334842	10	9093	-	38.77
ND2	Paris	Carbon storage regulator	1192799 - 1199183	10	6385	-	36.76
ND3	Paris	Nickel/Cobalt type II transport systems	1353408 - 1356362	6	2955	-	39.63
ND4	Philadelphia	No clear role	1439890 - 1450778	10	10889	+	35.17
ND5	Philadelphia	Mainly transposases and phages integrases	2892417 - 2904871	16	12455	+	34.97
ND6	Corby	No clear role	1182245 - 1189318	9	7074	-	36.78
ND7	Lens	Incomplete *HipA*/*HipB *system	2605322 - 2617191	12	11870	-	34.77
ND8	Paris	No clear role	1733202 - 1746135	7	12934	+	38.09
ND9	Paris	No clear role	2654264 - 2661604	9	7341	+	37.57
ND10	Paris	No clear role	2725059 - 2776774	61	51716	+	37
	Alcoy		3003085 - 3015471	28	12387	+	37.15
ND11		Mainly transposases					
	Corby		3443118 - 3451379	14	8262	+	35.00
ND12	Lens	No clear role	2824095 - 2849378	16	25284	-	36.77
ND13	Corby	No clear role	2833804 - 2842585	10	8782	+	37.42

First column describes the name of the island and the genome of provenance. Following columns report a short description, the position on the genomes, the number of CDSs contained in the island, the length, the presence (+)/absence (-) of transposase genes and the GC content.

#### Resistance-related islands (R)

We have identified two types of resistance-related islands, R1 and R2 (see Figure 4 and Figure 5, blue track). R1 maintains the same position, around 60 Kb at the beginning of the chromosome, in the five strains, inserted in a tRNA^asn ^site, although the organization and content is different. In Lpa, Lpc and Lpg, the island is similar and contains several hypothetical proteins as well as a methylase (lpa00094, LPC_0075, lpg0060), followed by a multi-drug resistance protein (lpa00095, LPC_0076, lpg0061). In the Paris strain, the island also begins with a methylase (lpp0063) and in the Lens strain with a putative transposase (lpl0064). However, although the position of the gene coding for the methylase is quite similar in both genomes, the alignment is different, due to a relatively big deletion in Lpl, whereas in Lpp it is followed by the antibiotic persistence-related system *HipB/HipA *(lpp0065, lpp0064) with no homolog in other *Legionella *strains. Although the mechanism of this system is still not well known, much evidence points to HipA as a toxin with bacteriostatic activity which binds DNA/RNA, blocking macromolecule synthesis until HipB binds HipA, releasing the DNA/RNA so microbial cells can survive extended exposure to drugs [22,23]. This island is followed by various phage-related proteins, elements of the Xre family of transcriptional regulators, an LvrA protein, and three transporters (lpp0077, lpp0078, lpp0079) of which the lpp0077 is similar to the acriflavine multi-drug efflux pump [24]. Finally, the island ends with three hypothetical proteins and an IS4-family transposase (lpp0083). Several genes such as the TraK, the LvrA-related protein and the phage-related integrases are also maintained in the same positions in the Alcoy, Corby, Lens and Philadelphia strains. A similar system (R2), was also found in the Lens strain. It is a small region containing transposases as well as two homologs of a stability system *StbDE *(lpl1587, lpl1588). This island was originally associated with plasmids [25], but it has also been found on the chromosomes of other pathogenic bacteria [26].

#### Transport/secretion systems (TS)

Only one TS island has been found in the Lpa and Lpc genomes. TS1 in the Alcoy strain is composed of 16 ORFs (lpa01590 to lpa01614). Three of these ORFs, lpa01601, lpa01599 and lpa01598, are related to the cobalt/zinc/cadmium efflux HelABC transport system that provides resistance against these heavy metals [27]. They are followed by lpa01604, which codes for the metallo-regulator ArsR that, in the presence of metals, derepresses the operator/promotor DNA, thereby activating the transcription of downstream genes [28]. As happens in other islands, this ends with a phage integrase and three transposase-related ORFs, indicating a possible exogenous origin in *Legionella*. It is worth mentioning that all five genomes carry a *Hel*ABC operon belonging to the core genome, while Alcoy and Corby strains also possess the two additional above-mentioned systems.

In the Corby strain, TS1 is bigger than in the Alcoy, spanning about 50 kb. It contains the previously mentioned *Hel*ABC operon (LPC_1847- LPC_1849), in addition to the *Hel*ABC systems present as part of the core genome (LPC_02269, LPC_02270, LPC_02271), a transposase (LPC_1856) and a phage repressor protein (LPC_1857). The first 9 ORFs of the TS1c island (mainly hypothetical proteins and one transposase) are syntenic with the Paris ND9 island (see below). The island continues for about 19 kb with apparently no synteny with other genomes and then regains synteny with the Paris ND10 island.

Interestingly, the TS1a-HelABC genes seem to be more similar to the core Lpl operon than to that of Lpc TS1 while TS1c-HelABC genes are more similar to those of the Lpg genome. Conversely, core HelABC genes in Lpa and Lpc are highly related.

#### DNA transfer-related islands (DT)

Three DT islands have been identified (DT1, DT2 and DT3). DT1 and DT2 correspond to the *Trb-2 *and *Trb-1 *islands described by Glöckner and collaborator for the Corby strain [7]. We have found that both islands are also present in the Alcoy strain, although DT2a is shorter than DT2c, thus suggesting that Lpa and Lpc acquired these systems via DNA transfer prior to their divergence (see Figure 5, green track). Some remarkable features of DT1 are: a phage repressor (lpa00219, LPC_0166), a set of *tra *and *trb *(conjugal transfer proteins) operons, a putative lamboid prophage *Rac *integrase (lpa00266, LPC_0199), another integrase (lpa00270, LPC_0202), an *htpX *protease (lpa00275, LPC_0205), and a prophage regulatory protein *alpA *(lpa00278, LPC_0208). In the DT2 island, both strains share a putative RNA helicase (lpa00835, LPC_2785), two putative restriction enzymes (LPC_2788, LPC_2790, lpa00832, and lpa_00829) and the set of the *tra *and *trb *operons. Glöckner and collaborators [7] described that Trb-1 (here DT2) is active and could be transferred to other *Legionella*. In both genomes, DT1 and DT2 are integrated, respectively, at the tmRNA and tRNA^pro ^sites.

Finally, we have identified a DNA transfer island, DT3, in all strains with the exception of the Corby and Alcoy ones. It is worth mentioning that this island was previously described in Lpg and Lpp, as an integrated plasmid-like element [5,29]. It contains *lvh *(*Legionella *vir homolog), a type IV secretion system involved in conjugation [30,31]. The *lvr *(Legionella vir region) is located downstream where LvrA is homologous to the CsrA repressor, important for the inhibition of post-exponential phase activity (such as DNA transfer) [32]. A CRISPR system was identified at the beginning of this island.

#### Clustered Regularly Interspaced Short Palindromic Repeats (CRISPR) systems (C)

CRISPR are bacteriophage resistance systems [33] that have been identified in the Alcoy and Lens (C1), and in Paris (C2) strains (Figure 5, pink track). Lpl also possesses one almost identical CRISPR system on the plasmid. Phylogenetic analyses reveal how Alcoy CAS (CRISPR-associated genes) genes are more closely related to the ones on the plasmidic CRISPR of the Lens strain than to the chromosomal one. The CAS genes identified were Cas1 (lpl2837, plpl0052, lpa01472), Cas3-helicase (plpl0051, lpl2838, lpa01473), and lpa01474 and lpa01475, which are two conserved proteins in bacteria, but with no relatives in other *Legionella *genomes. lpa01476, plpl0047 and lpl2842 belong to the CRISPR-associated Csy4 family proteins (see Additional file 2). Downstream of this cluster of proteins begins the repeats of the CRISPR system (see Additional file 3). The Alcoy and Lens strain repeats are almost identical, except for one base (adenine in the Alcoy strain and cytosine in the Lens, GTT(A/C)ACTGCCGCACAGGCAGCTTAGAA). Fifty-seven direct repeats were found in Lpa, while 53 in the Lpl plasmid, and two clusters of 52 and 12 on the Lpl chromosome. None of the Alcoy spacers were found to be similar to other spacers from the CRISPR database [34].

The Paris strain genome hosts a CRISPR system, located at the beginning of the DT3 island. The first CRISPR-related protein is coded by lpp0160 and shows a weak similarity to a putative CRISPR-associated large protein. The cluster is followed by Cas1 (lpp0161), Cas2 (lpp0162), Cas4 (lpp0163) and 34 direct repeats. Interestingly, the repeats and the CRISPR-associated genes are not related to the ones found in the Lpa and Lpl genomes.

#### Phage related islands (PR)

Up to seven different phage-related islands have been found. The Alcoy and Corby strains share PR1 and PR3 whereas all the other PR islands are strain-specific. PR1 in Lpa and Lpc are almost syntenic and could be considered an ancient infection that took place before the split of the two lineages; a related region is also present in Lpg genome. This island contains several phage-related proteins, several transposases belonging to the IS4 family, an MviN virulence factor (lpa01685, LPC_2173, lpg1087), as well as other not well-defined proteins containing DNA cleavage or binding related domains. The MvinN-related protein is additional, but not equivalent to the constitutive one present on the chromosomes (see Additional file 4) of the five strains (lpa0385, LPC_0506, lpg2635, lpl2560, and lpp2688). MvinN has been described as an important factor of virulence in *Salmonella typhimurium *and *Burkholderia pseudomallei *[35]. Although its role in pathogenicity is still not clear, this protein is a homolog of the proposed lipid II flippase protein [36,37] that has no virulence activity.

PR2 is specific to the Philadelphia strain and relies on a probable hot spot region. It contains six residual transposase-related ORFs followed by four hypothetical proteins that correspond to the beginning of the DT1ac islands.

PR3, as stated above, has been found only in the Corby and Alcoy strains. It begins with an ankyrin repeat-containing protein (LPC_1606, lpa03089). Ankyrin-domain-containing proteins have a eukaryotic origin and are related to intracellular trafficking. In *Legionella *ankyrin-containing proteins may be secreted by the Dot/Icm system and could play a role in intracellular bacterial replication [14,38]. The island also contains several hypothetical proteins (LPC_1607-LPC_1615, lpa03090-lpa03095) and a transposase (LPC_1616, lpa03096).

PR4 is specific to the Lens strain genome and spans around 29 kb. It seems to be a residual plasmid integrated into the chromosome, based on the presence of several proteins relating to DNA organization, such as lpl1068 containing SNF2-related protein domains that seem to be similar to helicases, and an Omp/MotB domain-containing protein (lpl1070) that may be related to structural flagella membrane proteins. Several phage-related proteins and transposases were found, together with another predicted inner membrane protein (lpl1073). Moreover, this island contains an ORF similar to DNA damage-inducible protein J (lpl1084), and the last two ORFs (lpl1092 and lpl1093) have high homology to other plasmid maintenance killer/antidote systems. The latter has been identified in several gram-negative-related plasmids and it is known as a regulator of bacterial programmed cellular death, although in some cases (e.g. *E. coli*) it is also integrated into the chromosome [39].

The Alcoy-specific island PR5 is integrated into a [CAT]-tRNA^ile ^site. The first three ORFs code for proteins related to transposases, whereas the remainder code for hypothetical proteins with no clear function. None of these proteins appear to have any relationship with other *Legionella*-related ORFs, and seem to be a clear case of acquisition by HGT. Three genes with no clear function, TraK gene (lpa03390), an inner membrane protein (lpa03394), and one hypothetical protein (lpa03395), have been found to be syntenic with the Lpg and Lpl genomes (lpg2365, lpg2366, and lpg2367; lpl0071, lpl0070, and lpl006, respectively). lpa03400 is similar to a phage-related integrase. The next ORFs (on the reverse strand) are related to a cluster of genes involved in bacilysin synthesis, known as one of the simplest antibiotic peptides active against some bacteria and fungi [40] (see Additional file 5).

PR6 in the Philadelphia genome includes homologs of a type IV secretion system, mobile genetic elements, and virulence factors. It has been described in detail by Brassinga and collaborator (2003) as a 65 kb pathogenicity island [41].

Finally, the island PR7 in the Paris genome codes almost exclusively for hypothetical proteins. Only a putative primase/helicase (lpp2117), and a phage integrase (lpp2123) homolog seems to relate this island to phage integration with an unpredicted function.

#### Not defined island (ND)

Up to thirteen islands, for which it has not been possible to establish a clear role, have been identified. Interestingly, all but one (ND11 in Lpa and Lpc), are strain-specific islands. ND1, ND2, ND3, ND8, ND9, and ND10 are found in the Paris genome. ND1 hosts a complete cytochrome *o *cluster (subunits II, I, III, IV on lpp0294, lpp0295, lpp0296, lpp0297, respectively) and a glycine/betaine transporter. ND2 contains hypothetical proteins, a CsrA (Carbon storage regulator), and is located close to the chromosomal heavy metals regulatory genes (*HelABC*). ND3 is formed by two hypothetical proteins followed by the HupE/UreJ membrane protein (lpp12118), homologs of Nickel/Cobalt type II transport systems [42]. These elements are followed by a thiocyanate hydrolase cluster for subunits gamma, alpha and beta (lpp1219, lpp1220 and lpp122, respectively) that were previously identified as unique to the Paris genome when compared to that from Lens [6]. This enzyme is the first key step in thiocyanate degradation and it is important in the detoxification processes [43]. ND8 is a small island composed mainly of transposases. Finally, for ND9 and ND10, it was not possible to propose a role. ND4 and ND5 are specific to the Philadelphia strains. ND4 is a small island containing hypothetical proteins and transposases, whereas ND5 contains a whole set of transposases and phage integrases as well as hypothetical proteins. ND6 and ND13 are specific to the Corby strain. ND6 spans a 7 kb region in the Corby genome with no apparently exogenous genes; it contains mainly hypothetical proteins, although some ORFs seem to be related to acetyltransferases. ND13 is syntenic with the terminal part of the Lpp island ND10 and, similarly, consists of hypothetical proteins. ND11 was found to be syntenic in both the Alcoy and Corby genomes and contains transposases and hypothetical proteins. ND7 and ND12 islands were found only in the Lens strains. ND7 is another not well-defined island consisting mostly of hypothetical proteins. It contains an ORF related to filamentation induced by a cAMP protein (lpl2288), an incomplete homolog of a HipA system, as well as the R1p island *Hip*A/*Hip*B system. Finally ND12 is made up exclusively of hypothetical proteins.

## Conclusions

The virulence and persistence of *L. pneumophila *are mainly due to specific mechanisms coded by part of its core genome that makes *L. pneumophila *able to infect, survive and replicate in macrophages [14,44-46]. Lpc has been described as one of the most virulent strains [47], while Lpp is responsible for sporadic cases but is frequently recognized worldwide [48]. Lpl was responsible for important outbreaks in France during 2003-2004 with 86 registered cases resulting in 17 deaths [6]. Finally, although the Lpg strain was the first one isolated for which the genome sequence was defined, it turned out to be not so virulent as the other [49].

Comparative genomics of five strains isolated in different parts of the world of *L. pneumophila *disclosed the presence of several HGT-related islands and an evident history of recombination events. Here, we reported a number of features connected to virulence that could have been exchanged, or acquired by the strains along with their evolution. The traces of these events are mainly part of the dispensable genome compartment.

The islands encountered from the dispensable pangenome compartment of the five genomes revealed factors that can give additional virulence to each strain. Alcoy and Corby strains are those in which more islands have been found related to virulence and DNA transfer activities. Multi-drug efflux systems have been found in Lpa, Lpc and Lpg, while stability systems have been found in Lpl and Lpp genomes (R1). Lpa and Lpc strains are probably potentially more resistant in the presence of heavy metals, due to an additional *HelABC *system in the TS1 island. Moreover, Lpa and Lpc seem to have acquired, before to their lineage split, the ability to be more successful in DNA transfer by the DT1ac and DT2ac systems. Interestingly, Alcoy strain also acquired a complete bacilysin system (PR5 island), probably by precedent phage contact after separation from the Corby lineage, which could represent an environmentally competitive advantage for this virulent strain. Lpa, Lpc the Lpg also carry an additional Mvin virulence factor, although there is no experimental evidence of its activity (island PR1). Moreover Alcoy, as well as Lens and Paris, proved to carry phage resistance systems (CRISPR on C1al and C2p islands). Several additional specific features have also been reported, although their role could not be predicted.

Finally, the data reported in this work show that the Alcoy strain possesses additional features, making it different from other previously sequenced genomes, even with the most closely related Corby strain. This finding could be related to the recurrent and sometimes mortal outbreaks recorded in the Spanish town of Alcoy.

## Methods

### Strains used in this work

*L. pneumophila *strain Alcoy 2300/99 was isolated from sputum of a patient with Legionnaires' disease (LD) and associated with the LD outbreak detected in Alcoy (Spain) in 1999. It belongs to the most predominant serogroup 1 [8]. The same strain was further isolated in other successive LD outbreaks in 2000 and 2002. The publicly available genomes of *L. pneumophila *used for comparison were retrieved from GenBank database http://www.ncbi.nlm.nih.gov/Genbank/index.html. Abbreviations and accession numbers are reported in Table 1.

### DNA extraction, shotgun clone libraries and sequencing

DNA from *L. pneumophila *Alcoy was extracted as described in D'Auria et al. (2008) [9]. Cloning and sequencing were carried out as follows: two libraries (inserts of 1-2 and 2-10 Kb) were generated by sonication of genomic DNA, followed by cloning of the fragments using the TOPO XL PCR Cloning Kit (Invitrogen, #K4700-10). Plasmid DNA purification was done with a Montage Plasmid Miniprep96 kit (Millipore, #LSKP09624) on a MULTIPROBE II-Robot Liquid Handling System (Packard Bioscience). Sequencing reactions were mainly performed using the ABI PRISM BigDye Terminator v3.0 Ready Reactions Kit and resolved using the 3730 Xl Genetic Analyzer (Applied Biosystems). To complete the assembly we used 454 pyrosequencing (Roche) performed on one half of a GS-FLX PicoTiter plate, obtaining a total of 52 Mb. The combination of both sequencing methods allowed the genome to be defined in 4 contigs. Finally, inverse PCR was employed to fill the remaining gaps and close the genome.

### Genome assembly and annotation

Base-calling of each Sanger read was carried out with the "Pregap4" interface from Staden Package [50]. All reads were then checked manually by the "Trev" program and the assembly of Sanger sequences was performed by Cap4 program, both from the Staden Package [50]. The 454 reads were assembled by the Newbler assembler http://www.roche-applied-science.com and then integrated with the previous Sanger assembly. Open reading frame predictions were carried out with the Glimmer3 program [51] assigning the "lpa" locus tag to each sequence. All CDSs were searched by BLAST [52] searches against the non-redundant GenBank database, the Cluster of Orthologous Groups [53] and the Kyoto Encyclopedia of Genes and Genomes [54]. Annotation was then improved by homology searches against previously sequenced genomes of the Philadelphia, Lens, Paris and Corby strains (see Table 1 for genome accession numbers). Ribosomal genes were identified by BLAST searches against "nt" databases. tRNAs were identified by the tRNAscanSE software [55]. tRNA genes with anticodon CAT (tRNAIle, tRNAMet and tRNAfMet) were identified by the method described by Silva and collaborators [56].

### Comparative analyses

CDSs from each genome were considered orthologous when reciprocal BLAST best hits gave at least 70% of overlap with a minimum of 80% similarity. A catalogue of orthologs was compiled. GenomeViz2 software was employed to draw genome plots [57]. Several Perl scripts were compiled in our laboratory for massive data handling (available upon request).

To define the coverage of the *L. pneumophila *pangenome, rarefaction curves were calculated from pools of CDS from each genome. In ecology, rarefaction is a technique applied in order to standardize and compare species richness computed from samples of different size [58]. Here, it is applied to compare gene cluster richness among multiple genomes from the same species. The *L. pneumophila *pangenome was then compared with pangenomes from strains belonging to *E. coli *(8 genomes), *Streptococcus pyogenes *(8 genomes), *Staphylococcus aureus *(9 genomes), *Streptococcus agalactiae *(8 genomes)(accession numbers are reported in Additional file 6). For each genome BLASTCLUST software was used to define gene clusters (70% similarity and 70% overlap). Gene abundance within each cluster was used to calculate rarefaction curves by the RarefactWin.exe http://www.uga.edu/~strata/software/Software.html program.

A comparative analysis among the five strains has been carried out using the Mauve, multiple genome alignment software [59].

### Pangenome distribution

All CDSs from each genome were pooled together and clustered by CD-HIT-EST software with at least 70% of overlapping and a minimum of 80% similarity [60]. One gene from each cluster was characterized by RPSBLAST best match (*e*-values lower than 10^-15^) against the COG database (Cluster of Orthologous Groups, [53]).

### Determination of specific islands

Discontinuity of the homology (synteny) between CDSs from a given genome and its ortholog in every comparison were considered to define an island. Generally, islands were defined when more than 5 consecutives CDSs were found to be specific for one strain. Syntenic Alcoy/Corby orthologous genes which did not match in the other three genomes were also considered islands. Islands were named according to their proposed function. A lowercase letter was added to the end of the name referring to the genome to which it belonged and letters were chosen according to the official locus tag definition ("a" for Alcoy, "c" for Corby, "g" for Philadelphia, "l" for Lens and "p" for Paris; e.g. "TS1a": Transport/Secretion island number 1 from the Alcoy genome). Due to the fact that the original annotations of Lpc, Lpg, Lpl and Lpp genomes often report CDSs as "hypothetical protein", similarity searches of genes within these islands were carried out against an updated Refseq (GenBank) database http://www.ncbi.nlm.nih.gov/RefSeq/.

## Authors' contributions

GD participated in conception, genome assembly, comparative analysis, and drafted the manuscript. NJH participated in sequencing and genome assembly FPB participated in sequencing and genome assembly and annotation. AM participated in the conception and design of the study and critically revised the manuscript. AL participated in the conception, design and discussion of the study and critically revised the manuscript. All authors have read and approved the final manuscript.

## Supplementary Material

Additional file 1 **Presence/absence of islands among the five considered genomes**. The presence or absence of a given island (column headers) is reported for every genome (line). Orange island is common to all strain, blue islands are strain-specific islands, red islands are common to Lpa and Lpc genomes, the green island is present in Lpp, Lpg and Lpl but not in Lpa or Lpc genomes. For the other is not possible to make a clear hypothesis about their origin. Click here for file

Additional file 2 **CRISPR associated gene trees**. Rooted trees obtained by neighbor joining method applying Kimura distance. In bold the *Legionella pneumophila *str. Alcoy sequence. Relative sequences represent best hits from GenBank protein refseq database. Click here for file

Additional file 3 **CRISPR repeats structure in Alcoy genome**. Rooted trees obtained by neighbor joining method applying Kimura distance. In bold the *Legionella pneumophila *str. Alcoy sequence. Relative sequences represent best hits from GenBank protein Refseq database. Click here for file

Additional file 4 **Mvin gene trees**. Rooted trees obtained by neighbor joining method applying Kimura distance. In bold the *Legionella pneumophila *str. Alcoy sequence. Relative sequences represent best hits from GenBank protein Refseq database. Click here for file

Additional file 5 **Bacilysin containing island from Alcoy genome**. Bacilysin cluster begins with an ORF homolog of a phospho-2-dehydro-3-deoxyheptonate aldolase, which is an intermediate of the synthesis of chorismate (lpa03410); the next four ORFs are homologs of bacilysin biosynthesis BacA (lpa03409), BacB (lpa03408), BacC (lpa03407) and BacC (lpa03406); lpa03405 is a transporter of the multidrug/metabolite exporter family; lpa0304 is a purine metabolism-related protein. lpa03404 and lpa3403 are related to chorismate mutase and the subsequent amino-transferase could be related to the final steps in bacilysin biosynthesis [61]. Located further along the island, lpa03412 is a homolog of an S24-like peptidase, followed by two hypothetical proteins and a bacteriocin adenylyltransferase (lpa03416). The PR5 island ends with an IS10-related transposase, lpa03419, two hypothetical proteins and a phage related integrase (lpa03425). This island demonstrates evidence of cluster acting in bacilysin-like bacteriocin production that is specific to the Alcoy genome. Click here for file

Additional file 6 **Genomes used for rarefaction analysis**. For each organisms strain names and GenBank accession numbers are reported. Click here for file
