# Supplementary material for: Legionella pneumophila pangenome reveals strain-specific virulence factors
Source: BMC Genomics. 2010 Mar 17;11:181. doi: 10.1186/1471-2164-11-181 (PMC2859405; doi:10.1186/1471-2164-11-181)
Supplement: Additional file 2 — CRISPR associated gene trees. Rooted trees obtained by neighbor joining method applying Kimura distance. In bold the Legionella pneumophila str. Alcoy sequence. Relative sequences represent best hits from GenBank protein refseq database. [file 1471-2164-11-181-S2.PPT]

## Slide 1
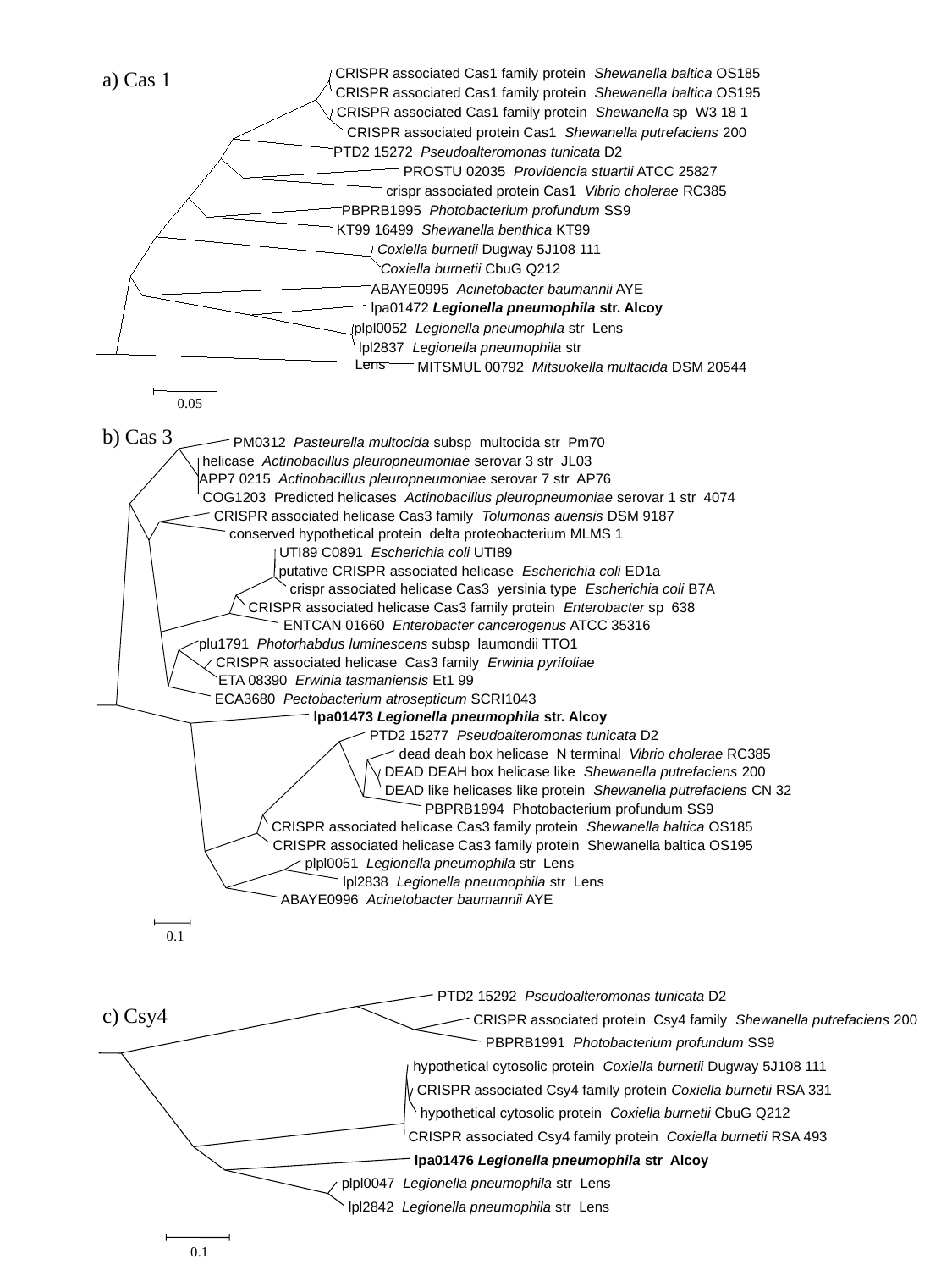

a) Cas 1
 CRISPR associated Cas1 family protein Shewanella baltica OS185
 CRISPR associated Cas1 family protein Shewanella baltica OS195
 CRISPR associated Cas1 family protein Shewanella sp W3 18 1
 CRISPR associated protein Cas1 Shewanella putrefaciens 200
PTD2 15272 Pseudoalteromonas tunicata D2
 PROSTU 02035 Providencia stuartii ATCC 25827
 crispr associated protein Cas1 Vibrio cholerae RC385
PBPRB1995 Photobacterium profundum SS9
 KT99 16499 Shewanella benthica KT99
 Coxiella burnetii Dugway 5J108 111
Coxiella burnetii CbuG Q212
ABAYE0995 Acinetobacter baumannii AYE
 lpa01472 Legionella pneumophila str. Alcoy
plpl0052 Legionella pneumophila str Lens
 lpl2837 Legionella pneumophila str Lens
 MITSMUL 00792 Mitsuokella multacida DSM 20544
0.05
b) Cas 3
 PM0312 Pasteurella multocida subsp multocida str Pm70
 helicase Actinobacillus pleuropneumoniae serovar 3 str JL03
APP7 0215 Actinobacillus pleuropneumoniae serovar 7 str AP76
 COG1203 Predicted helicases Actinobacillus pleuropneumoniae serovar 1 str 4074
 CRISPR associated helicase Cas3 family Tolumonas auensis DSM 9187
 conserved hypothetical protein delta proteobacterium MLMS 1
 UTI89 C0891 Escherichia coli UTI89
 putative CRISPR associated helicase Escherichia coli ED1a
 crispr associated helicase Cas3 yersinia type Escherichia coli B7A
 CRISPR associated helicase Cas3 family protein Enterobacter sp 638
 ENTCAN 01660 Enterobacter cancerogenus ATCC 35316
plu1791 Photorhabdus luminescens subsp laumondii TTO1
 CRISPR associated helicase Cas3 family Erwinia pyrifoliae
ETA 08390 Erwinia tasmaniensis Et1 99
 ECA3680 Pectobacterium atrosepticum SCRI1043
 lpa01473 Legionella pneumophila str. Alcoy
 PTD2 15277 Pseudoalteromonas tunicata D2
 dead deah box helicase N terminal Vibrio cholerae RC385
 DEAD DEAH box helicase like Shewanella putrefaciens 200
 DEAD like helicases like protein Shewanella putrefaciens CN 32
 PBPRB1994 Photobacterium profundum SS9
 CRISPR associated helicase Cas3 family protein Shewanella baltica OS185
 CRISPR associated helicase Cas3 family protein Shewanella baltica OS195
 plpl0051 Legionella pneumophila str Lens
 lpl2838 Legionella pneumophila str Lens
ABAYE0996 Acinetobacter baumannii AYE
0.1
c) Csy4
 PTD2 15292 Pseudoalteromonas tunicata D2
 CRISPR associated protein Csy4 family Shewanella putrefaciens 200
 PBPRB1991 Photobacterium profundum SS9
 hypothetical cytosolic protein Coxiella burnetii Dugway 5J108 111
 CRISPR associated Csy4 family protein Coxiella burnetii RSA 331
 hypothetical cytosolic protein Coxiella burnetii CbuG Q212
 CRISPR associated Csy4 family protein Coxiella burnetii RSA 493
 lpa01476 Legionella pneumophila str Alcoy
 plpl0047 Legionella pneumophila str Lens
 lpl2842 Legionella pneumophila str Lens
0.1
