# Supplementary figures and images for: Legionella pneumophila pangenome reveals strain-specific virulence factors
Source: BMC Genomics. 2010 Mar 17;11:181. doi: 10.1186/1471-2164-11-181 (PMC2859405; doi:10.1186/1471-2164-11-181)

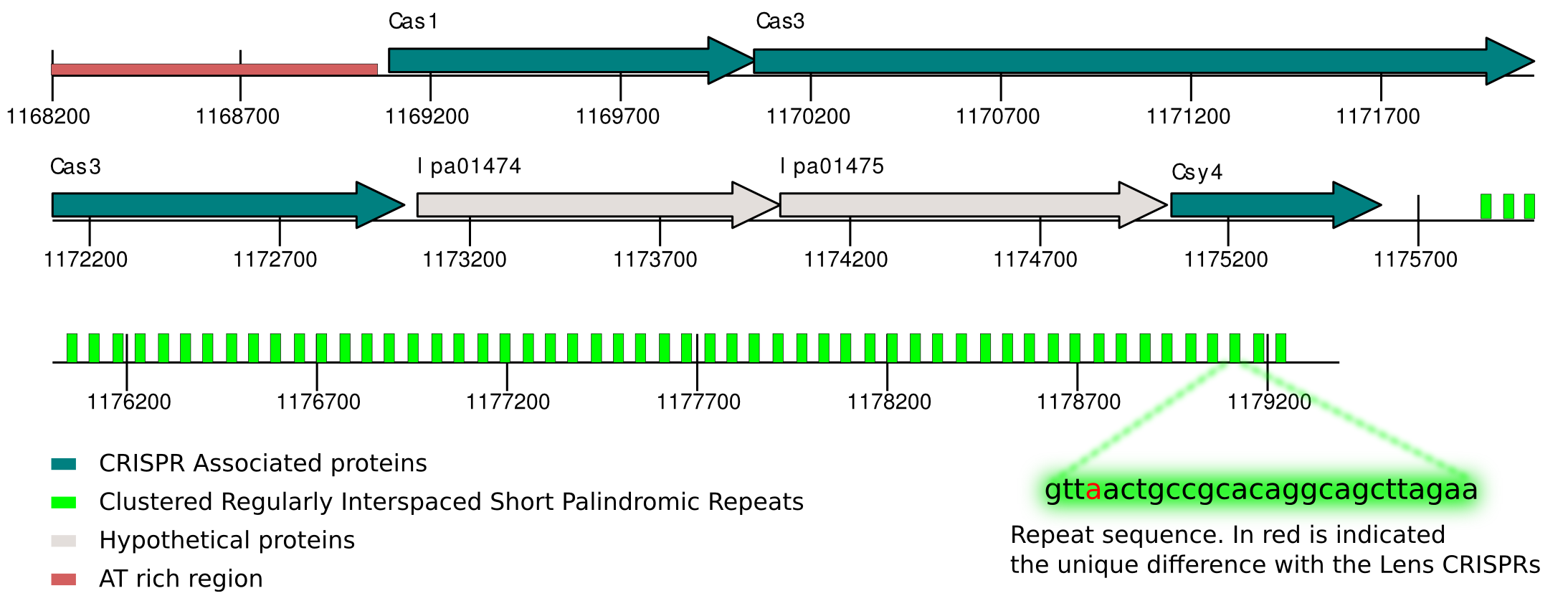

Supplement: Additional file 3 — CRISPR repeats structure in Alcoy genome. Rooted trees obtained by neighbor joining method applying Kimura distance. In bold the Legionella pneumophila str. Alcoy sequence. Relative sequences represent best hits from GenBank protein Refseq database. [file 1471-2164-11-181-S3.PNG]

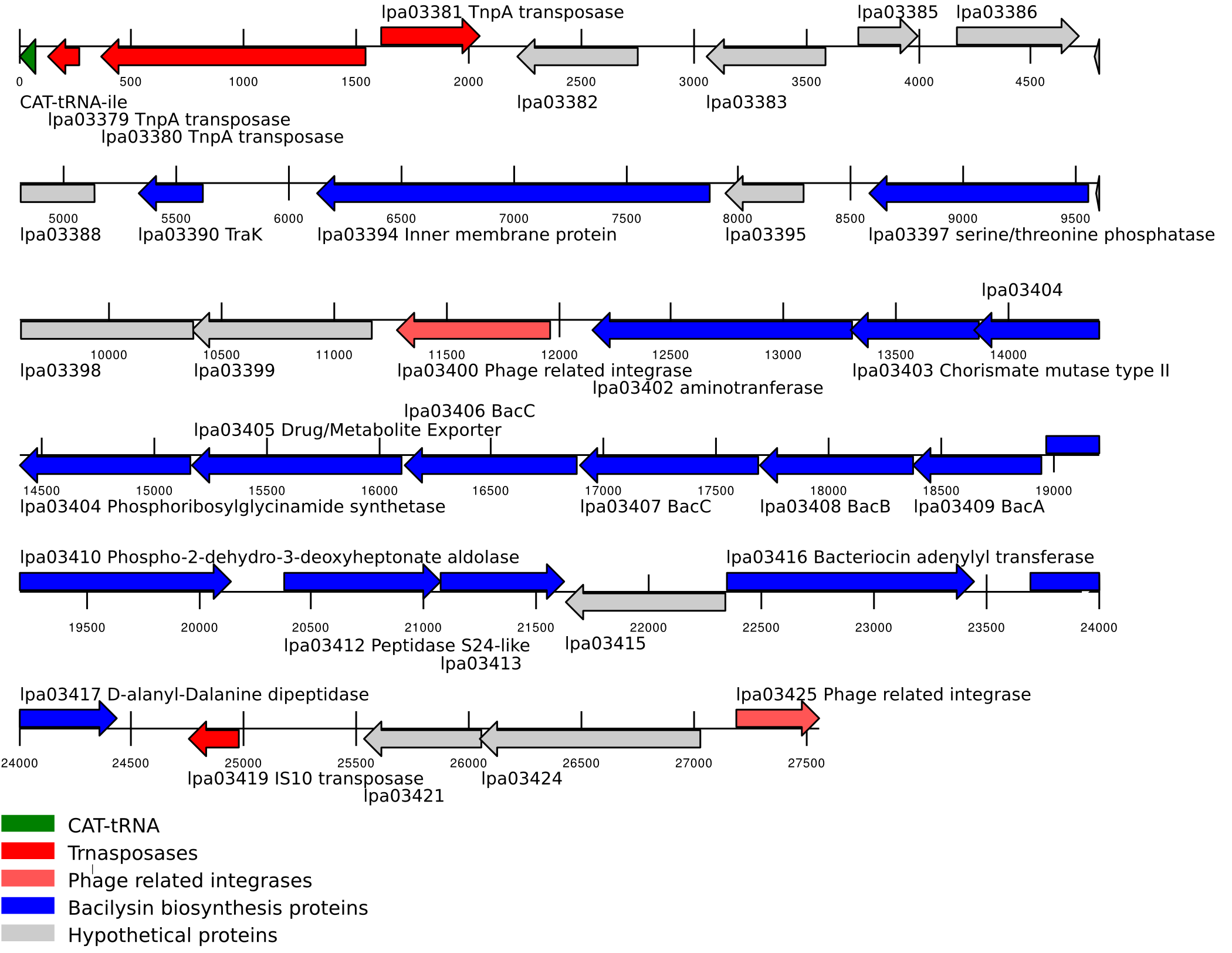

Supplement: Additional file 5 — Bacilysin containing island from Alcoy genome. Bacilysin cluster begins with an ORF homolog of a phospho-2-dehydro-3-deoxyheptonate aldolase, which is an intermediate of the synthesis of chorismate (lpa03410); the next four ORFs are homologs of bacilysin biosynthesis BacA (lpa03409), BacB (lpa03408), BacC (lpa03407) and BacC (lpa03406); lpa03405 is a transporter of the multidrug/metabolite exporter family; lpa0304 is a purine metabolism-related protein. lpa03404 and lpa3403 are related to chorismate mutase and the subsequent amino-transferase could be related to the final steps in bacilysin biosynthesis [61]. Located further along the island, lpa03412 is a homolog of an S24-like peptidase, followed by two hypothetical proteins and a bacteriocin adenylyltransferase (lpa03416). The PR5 island ends with an IS10-related transposase, lpa03419, two hypothetical proteins and a phage related integrase (lpa03425). This island demonstrates evidence of cluster acting in bacilysin-like bacteriocin production that is specific to the Alcoy genome. [file 1471-2164-11-181-S5.PNG]
