# Supplementary material for: Legionella pneumophila pangenome reveals strain-specific virulence factors
Source: BMC Genomics. 2010 Mar 17;11:181. doi: 10.1186/1471-2164-11-181 (PMC2859405; doi:10.1186/1471-2164-11-181)
Supplement: Additional file 4 — Mvin gene trees. Rooted trees obtained by neighbor joining method applying Kimura distance. In bold the Legionella pneumophila str. Alcoy sequence. Relative sequences represent best hits from GenBank protein Refseq database. [file 1471-2164-11-181-S4.PPT]

## Slide 1
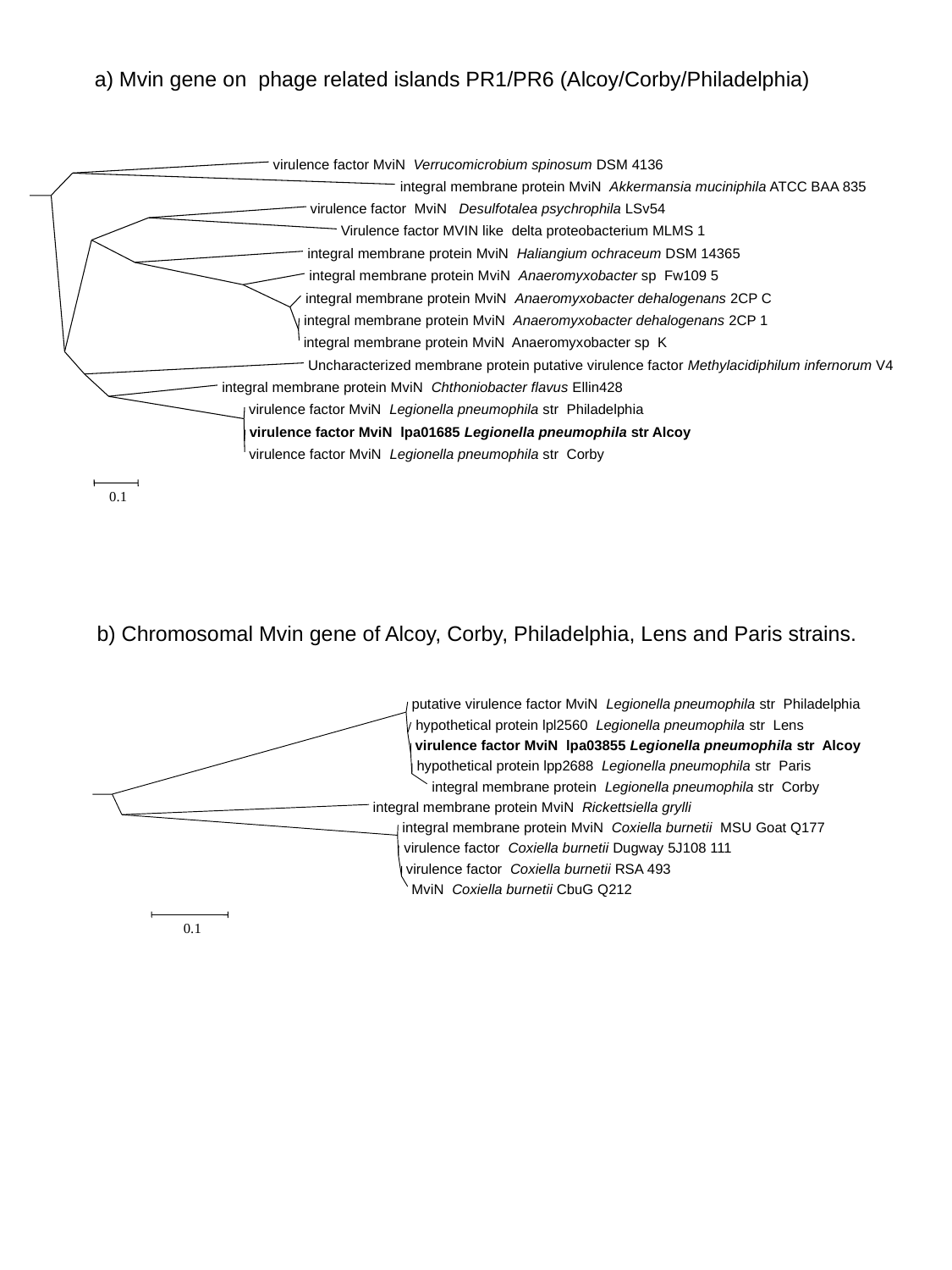

a) Mvin gene on phage related islands PR1/PR6 (Alcoy/Corby/Philadelphia)
 virulence factor MviN Verrucomicrobium spinosum DSM 4136
 integral membrane protein MviN Akkermansia muciniphila ATCC BAA 835
 virulence factor MviN Desulfotalea psychrophila LSv54
 Virulence factor MVIN like delta proteobacterium MLMS 1
 integral membrane protein MviN Haliangium ochraceum DSM 14365
 integral membrane protein MviN Anaeromyxobacter sp Fw109 5
 integral membrane protein MviN Anaeromyxobacter dehalogenans 2CP C
 integral membrane protein MviN Anaeromyxobacter dehalogenans 2CP 1
 integral membrane protein MviN Anaeromyxobacter sp K
 Uncharacterized membrane protein putative virulence factor Methylacidiphilum infernorum V4
 integral membrane protein MviN Chthoniobacter flavus Ellin428
 virulence factor MviN Legionella pneumophila str Philadelphia
 virulence factor MviN lpa01685 Legionella pneumophila str Alcoy
 virulence factor MviN Legionella pneumophila str Corby
0.1
b) Chromosomal Mvin gene of Alcoy, Corby, Philadelphia, Lens and Paris strains.
 putative virulence factor MviN Legionella pneumophila str Philadelphia
 hypothetical protein lpl2560 Legionella pneumophila str Lens
 virulence factor MviN lpa03855 Legionella pneumophila str Alcoy
 hypothetical protein lpp2688 Legionella pneumophila str Paris
 integral membrane protein Legionella pneumophila str Corby
 integral membrane protein MviN Rickettsiella grylli
 integral membrane protein MviN Coxiella burnetii MSU Goat Q177
 virulence factor Coxiella burnetii Dugway 5J108 111
 virulence factor Coxiella burnetii RSA 493
 MviN Coxiella burnetii CbuG Q212
0.1
