# Supplementary material for: Legionella pneumophila pangenome reveals strain-specific virulence factors
Source: BMC Genomics. 2010 Mar 17;11:181. doi: 10.1186/1471-2164-11-181 (PMC2859405; doi:10.1186/1471-2164-11-181)
Supplement: Additional file 6 — Genomes used for rarefaction analysis. For each organisms strain names and GenBank accession numbers are reported. [file 1471-2164-11-181-S6.DOC]

**Additional file 6**

Genomes used for rarefaction analysis. For each organisms strain names and GenBank accession numbers are reported

| *Escherichia coli* | | *Staphylococcus aureus* | | *Streptococcus pyogenes* | | *Streptococcus agalactiae* | |
| --- | --- | --- | --- | --- | --- | --- | --- |
| Strain | Accession  number | Strain | Accession  number | Strain | Accession  number | Strain | Accession  number |
| K12-W3110 | AP009048 | NCTC-8325 | CP000253 | M1-GAS | AE004092 | CJB111 | AAJQ00000000 |
| 0157:H7-Sakai | BA000007 | N315 | BA000018 | MGAS10750 | CP000262 | 2603V/R | AE009948 |
| K12-MG1655 | U00096 | MW2 | BA000033 | MGAS2096 | CP000261 | A909 | CP000114 |
| UTI89 | CP000243 | MSSA476 | BX571857 | MGAS10270 | CP000260 | 18RS21 | AAJO00000000 |
| APEC-01 | CP000468 | COL | CP000046 | MGAS6180 | CP000056 | 515 | AAJQ00000000 |
| 0157:H7EDL933 | AE005174 | USA300 | CP000730 | MGAS5005 | CP000017 | H36B | AAJS00000000 |
| CFT073 | AE014075 | RF122 | AJ938182 | MGAS10394 | CP000003 | NEM316 | AL732656 |
| 536 | CP000247 | Mu50 | BA000017 | SSI-1 | BA000034 | COH1 | AAJR00000000 |
|  |  | MRSA252 | BX571856 |  |  |  |  |
